# Cellular Myxoma of the Vocal Cord: A Case Report and Review of the Literature

**DOI:** 10.5146/tjpath.2017.01417

**Published:** 2020-01-15

**Authors:** J. Fernando Val-Bernal, María Martino, M. Yolanda Longarela

**Affiliations:** Pathology Unit, Medical and Surgical Sciences Department, University of Cantabria and Idival, Santander, Spain; Anatomical Pathology Service, Marqués de Valdecilla University Hospital, Medical Faculty, University of Cantabria and Idival, Santander, Spain; Ear, Nose, and Throat Service, Marqués de Valdecilla University Hospital and Idival, Santander, Spain

**Keywords:** Larynx, Vocal cord, Myxoma, Cellular myxoma, Myxoid sarcoma

## Abstract

Myxomas are rare in the vocal cords. A 69-year-old man was admitted with one-year history of progressive dysphonia. Laryngoscopy revealed a polypoid mass on the right vocal cord. The diagnosis was cellular myxoma. A review of the literature including the present case revealed eleven reported cases of myxoma. Ten cases were classic myxoma. To the best of our knowledge, cellular myxoma has not been previously reported in the vocal cord. Hypercellularity does not affect the behavior of cellular myxoma. However, its recognition is important to prevent confusion with the group of low-grade myxoid sarcomas. Cellular myxoma should be considered in the differential diagnosis of any vocal cord mass.

## INTRODUCTION

Classic myxoma is a benign mesenchymal paucicellular tumor characterized by bland spindle and stellate shaped cells embedded in hypovascular, abundant loose myxoid stroma. The cellular variant of this tumor shows hypercellularity, more numerous collagen fibers, and increased vascularity (1,2).

Myxomas of the larynx are very uncommon. The reported sites of involvement are the vocal cords, the aryepiglottic fold, and the epiglottis. They are more common in the vocal cords. As far as we are aware, only ten cases of vocal cord myxomas have been previously reported. We describe herein a case of the cellular variant of myxoma in the right vocal cord and review the literature. To the best of our knowledge, a cellular myxoma (CM) in a vocal cord has not been previously reported.

## CASE REPORT

A 69-year-old man was admitted to the hospital with one-year history of progressive dysphonia. There was no associated pain, stridor, hemoptysis or weight loss. He was currently consuming 40 g of alcohol a day and had quit smoking 20 years ago. The patient was diagnosed with a polyp of the right vocal cord 20 years ago, but he refused the surgical treatment. Medical history was also significant for atrial fibrillation with multiple embolisms in the vertebrobasilar artery, brachiocephalic trunk, and mesenteric artery and chronic hepatic disease with thrombosis of the right portal vein. Syndromic associations were not present. Flexible laryngoscopy revealed a large polypoid lesion on the right cord with preserved mobility.

The patient underwent phonosurgery under general anesthesia.

**Figure 1:**
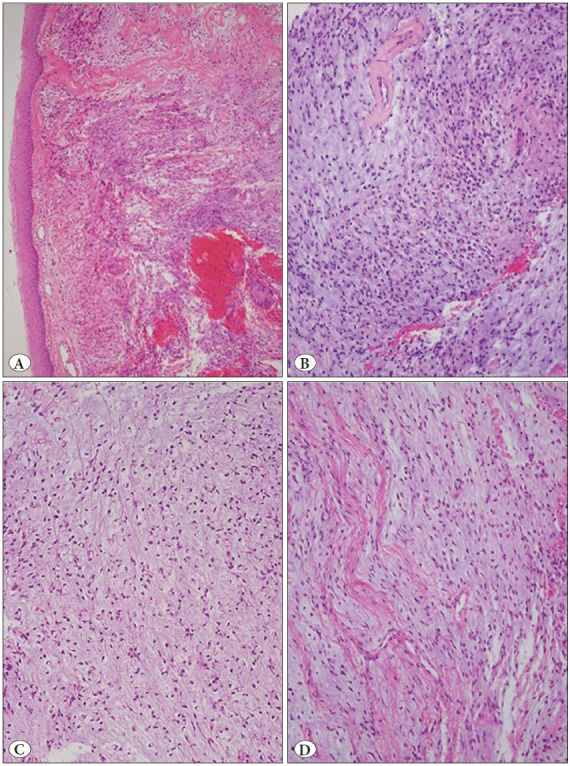
**A)** Vocal cord lesion showing a squamous mucosa. Submucosa is occupied by a cellular myxomatous neoplasm (H&E; x100). **B)** Area with variable cell density (H&E; x200). **C)** Hypocellular and hypovascular area (H&E; x200). **D)** Cellular area containing increased collagen fibers and vessels (H&E; x200).

**Figure 2:**
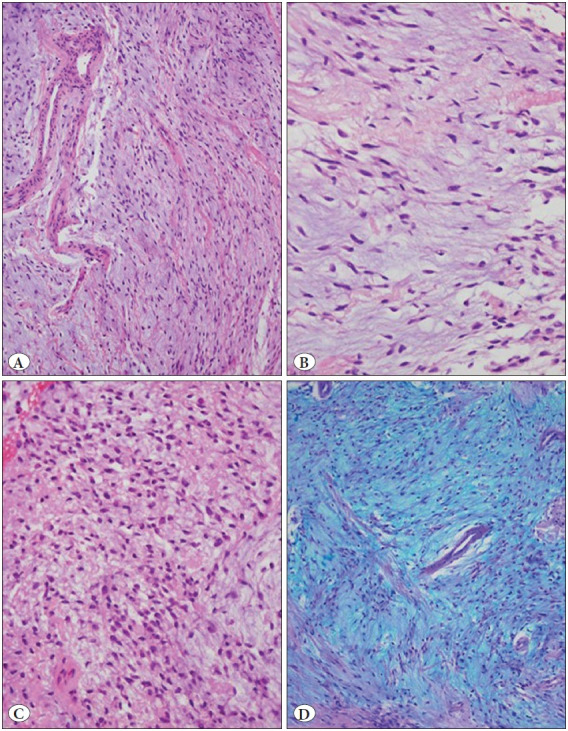
**A)** Prominent vessels, some of which are thick-walled containing smooth muscle (H&E; x200). **B)** Uniform and cytologically bland spindle cells in a moderately hypercellular region. Tumor cells are separated by mucoid matrix and generally do not touch one another (H&E; x400). **C)** High magnification appearance of a hypercellular area. Nuclei are uniform and pyknotic with tapered cytoplasms. Cells lack nuclear atypia (H&E; x400). **D)** Cells are suspended in abundant mucoid material that stains positively with Alcian blue at pH 2.5. A thick-walled vessel can be seen in the center of the image (Alcian blue; x200).

**Figure 3:**
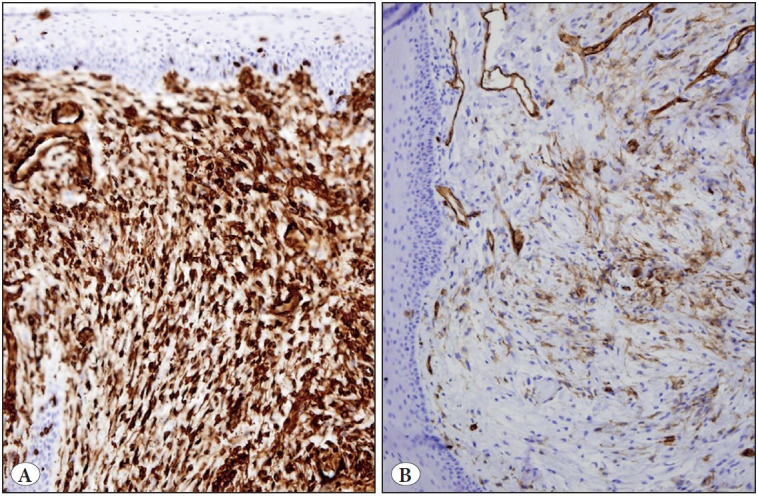
**A)** Hypercellular region showing diffuse reactivity for vimentin (IHC; x200). **B)** Hypocellular region showing focal positivity for CD34 (IHC; x200).

The specimen consisted of a glistening white, gelatinous, polypoid mass measuring 0.9 x 0.6 x 0.3 cm. Histopatho-logical examination revealed an excrescent tissue fragment consisting of squamous mucosa that was partially atrophic and a mesenchymal neoplasm (Figure 1). The tumor showed spindled and stellate cells suspended in a background of loose myxoid matrix. Cell density was variable throughout the tumor with hypercellular (Figure 1) and hypocellular areas (Figure 1). Hypercellular areas occupied about 90% of the tumor. In these areas, there were more numerous blood vessels and collagen fibers (Figure 1). In addition, occasional thick-walled vessels with smooth muscle in their walls were present (Figure 2). Tumor cells were uniform and bland in appearance (Figure 2). They showed small hyperchromatic nuclei with scant tapering eosinophilic cytoplasm (Figure 2). Scattered muciphages were also observed. Fluid-filled microcystic spaces were seen occasionally. Cellular pleomorphism, multinucleated giant cells, mitoses, or necrosis were not present. The myxoid matrix stained positive with Alcian blue at pH 2.5 (Figure 2). Immunohistochemical study revealed diffuse positivity for vimentin (Figure 3) and focal positivity for CD34 (Figure 3) in the constituent cells. These cells were not reactive for S100 protein, neurofilament protein, epithelial membrane antigen, claudin-1, GLUT-1, smooth muscle actin and MUC4. Ki-67 labeled only a few nuclei of the squamous epithelium. The deep surgical border was very close to the tumor boundary.

The patient was discharged in hours. One month later his voice was much improved. No signs of recurrence were observed.

## DISCUSSION

Pure myxomas are very infrequent in the vocal cords. A review of the literature revealed only ten previously reported cases (Table 1) (3–12). Mean patient age is 58.2 years (SD 13.5; range 36-77 years). The tumor is more frequent in males (male: female, 4.5:1). The main complaint varies from hoarseness or dysphonia to dyspnea (Table 1). The neoplasm can present with life-threatening dyspnea requiring tracheotomy (5,11). One case showing sleep apnea was cured after removal of the tumor (6). The average maximum diameter of the tumor was 1.03 cm (SD 0.62; range 0.4-2.5 cm). The majority of the lesions are located on the right vocal cord (2:1). Ten cases were classic myxoma. The present case was a CM. As far as we are aware, a CM has not been reported in the vocal cord. Excision of vocal cord myxoma is considered curative. In one case, removal of the neoplasm was incomplete. Recurrence is possible in theory but it has never been reported (Table 1).

**Table 1:** Vocal cord myxomas reported in literature

**Case/Reference**	**Age (years) /Sex**	**Main complaint**	**Site**	**Size** **(cm)**	**Type**	**Removal**	**Recurrence**
1/[3]	64/M	Dysphonia	Left	1.0	Classic	Incomplete	None
2/[4]	57/M	Hoarseness	Right	0.7	Classic	Complete	None
3/[5]	62/M	Dyspnea	Right	2.5	Classic	Complete	Not reported
4/[6]	42/M	Dyspnea, sleep apnea	Both	Not reported	Classic	Complete	None
5/[7]	46/M	Hoarseness	Right	0.8	Classic	Complete	Not reported
6/[8]	74/F	Hoarseness	Right	0.4	Classic	Complete	None
7/[9]	48/F	Dysphonia	Left	Not reported	Classic	Complete	None
8/[10]	36/M	Hoarseness	Right	0.7	Classic	Complete	None
9/[11]	65/M	Dyspnea	Right	1.5	Classic	Complete	None
10/[12]	77/M	Hoarseness/Dysphonia	Left	0.8	Classic	Complete	None
11/Present report	69/M	Dysphonia	Right	0.9	Cellular	Complete	None

Cells of a myxoma originate from modified fibroblastic cells that lack the ability to polymerize collagen. As an alternative, they produce an excessive amount of glycosaminoglycans giving them a gelatinous appearance on gross examination. The process suggests an underlying localized error in tissue metabolism (13).

CM is characterized by hypercellular areas that occupy from 10 to 90% of the tumor. These foci have increased number of cells, more prominent vascularity, increased collagen content and less extracellular myxoid matrix than classic myxoma. The hypercellular regions are not associated with cytologic atypia, multinucleated giant cells, mitotic activity, or necrosis. Vessels are capillary-sized but occasional thick-walled vessels with smooth muscle in their walls can be present. CMs usually show sparse paucicellular areas of classic myxoma with scant capillary-sized vessels (1,2).

All the cases of CM reported out of the larynx have behaved in a benign fashion with only a small risk of local non-destructive recurrence if not excised completely (1,2). Thus, in general, simple complete local excision is the adequate treatment.

The main differential diagnosis includes myxoid neurofibroma, low-grade myxofibrosarcoma, low-grade fibromyxoid sarcoma, and myxoid liposarcoma. Myxoid neurofibroma shows spindled elongated cells with tapering, wavy or bent nuclei and pale indistinct cytoplasms embedded in abundant myxoid background. Intralesional neural fibers are demonstrated with neurofilament protein. Besides, a considerable number of cells are positive for S100 protein (14,15). CM, unlike low-grade myxofibrosarcoma, does not show any cytonuclear atypia and does not have the classical curvilinear vascular architecture, often with a perivascular increase of cellularity (16). Low-grade fibromyxoid sarcoma is diffusely more cellular and is characterized by alternating myxoid and collagenous zones containing bland spindle cells with a whorled growth pattern. It may show areas of hyalinizing spindle cells with giant rosettes. MUC4 immunostaining has been found to be highly sensitive and specific for the diagnosis (17). Myxoid liposarcoma has small, bland spindle-shaped or more rounded cells, lipoblasts and a typically delicate plexiform or branching “chicken-wire” capillary vasculature (18).

In conclusion, CM of the vocal cord is a benign mesen-chymal tumor that shows foci of increased cellularity and vascularity, with presence of thick-walled vessels, and increased collagen content. The recognition of this tumor is important to avoid a misdiagnosis of any type of low-grade myxoid sarcoma. Although very rare, CM should be considered in the differential diagnosis of any vocal cord mass to allow for adequate treatment. Surgery is considered curative.
